# Changes in splenic volumes following stereotactic ablative radiotherapy (SABR) to adrenal tumors^☆^

**DOI:** 10.1016/j.ctro.2025.101011

**Published:** 2025-07-07

**Authors:** Nicolas Giraud, Miguel A. Palacios, John R. van Sornsen de Koste, Antonio M. Marzo, Peter S.N. van Rossum, Famke L. Schneiders, Suresh Senan

**Affiliations:** a Amsterdam UMC Location Vrije Universiteit Amsterdam, Department of Radiation Oncology, De Boelelaan 1117, Amsterdam, the Netherlands; b Cancer Center Amsterdam, Amsterdam, the Netherlands

**Keywords:** Adrenal metastases, MR-guided radiotherapy, IGRT, Spleen, SABR

## Abstract

**Purpose:**

Splenic irradiation can result in life-threatening infections. Updated dose constraints have been recommended for patients undergoing chemoradiotherapy and conventional radiotherapy but splenic constraints were not specified in trials of stereotactic ablative radiotherapy (SABR). We studied splenic doses in patients undergoing SABR for adrenal metastases and late changes in splenic volume (SV).

**Material and Methods:**

Patients treated with breath-hold MR-guided SABR for adrenal metastases were identified from an Ethics-approved database. Splenic dose constraints were not routinely used. The spleen was delineated retrospectively on both breath-hold CT and MR-scans. Mean spleen dose (MSD) and relative V_5-10-20-30Gy_ values were derived from the baseline plan. SV was measured on available follow-up CT scans at 6–12–24 months. Regression analyses were performed to assess SV changes in relation to splenic dose and other parameters.

**Results:**

SABR was delivered to 113 adrenal tumors mostly using 5 fractions (64 % of tumors), 3 fractions (19 %) or a single fraction (14 %). Systemic therapy was administered during or within 3 months preceding/after SABR in 51 % of patients. Left-sided tumors comprised 56 % of total, and baseline median MSD and V_10Gy_ were 9.7 Gy (range 1.5–28.4 Gy) and 46.3 % (range 0–100 %), respectively. Corresponding values for right-sided adrenal plans were 1.5 Gy (0.2–5.9 Gy) and 0 % (0–6.2 %), respectively. In multivariable analysis, a higher MSD was significantly associated with left laterality (p < 0.001), higher prescription dose (p = 0.02), and larger GTV (p < 0.001). An MSD of > 10 Gy was observed in 28 patients (25 %). Among these, a greater than 20 % decrease in SV was found in 46 % of patients with available follow-up at 6 months (n = 59), 40 % at 12 months (n = 47) and 50 % at 24 months (n = 31).

**Conclusion:**

Substantial reductions in spleen volume occur in 40–50 % of patients treated with adrenal SABR with an MSD of >10 Gy. The clinical relevance of splenic atrophy merits further study.

## Highlights

• Reductions in splenic volume at 6/12/24 months after adrenal SABR were observed in 40–50 % with mean spleen dose >10 Gy.
• Baseline splenic volume, mean spleen dose, spleen V5-10Gy were associated with a >20 % spleen volume reduction at 6 months.
• Re-planning with spleen dose optimization was feasible and still meeting other organs at risks’ institutional constraints.

## 1 Introduction

The increasing use of stereotactic ablative radiotherapy (SABR) for oligometastatic disease underscores the need for reliable organ-at-risk dose constraints. SABR protocols have generally not specified dose constraints for the spleen [[1], [2], [3], [4], [5]], but splenic irradiation during conventional radiotherapy is associated with volume loss, cytopenia (lymphopenia), and increased mortality due to sepsis [[6], [7], [8], [9], [10], [11], [12], [13], [14]]. Our magnetic resonance (MR-)guided SABR program commenced in 2016 before guidelines from the Royal College of Radiologists and the International Society of Paediatric Oncology (SIOP) recommended use of a mean spleen dose (MSD) < 10 Gy for conventional radiotherapy [15,16]. We retrospectively studied splenic doses during breath-hold MR-SABR for adrenal metastases, and assessed changes in post-SABR spleen volume (SV).

## 2 Material and methods

### 2.1 Patient selection and data collection

An ethics-approved institutional database (IRB00002991) was accessed to retrieve patient data on MR-SABR treatment for adrenal metastases using MRIdian systems (Viewray, Inc.) between 09/2016 and 01/2023. Collected details included patient demographics, ECOG performance status, histology, timing of metastatic disease, and systemic treatments.

### 2.2 Details of adrenal MR-SABR plans

Our MR-SABR approach has been described previously [17]. A breath-hold CT with 2.5 mm slice thickness and a 0.35 T MR with 0.16x0.16x0.3 cm resolution were acquired during shallow-inspiration breath-hold. The gross tumor volume (GTV) encompassed the entire affected adrenal gland. Planning target volume (PTV) was generated by adding a 3 mm isotropic margin. On-couch breath-hold MR-scans for daily plan adaptation were performed at each treatment fraction [18]. Treatment delivery was performed with breath-hold gating and IMRT step-and-shoot technique. Splenic dose constraints were not routinely used for planning.

### 2.3 SV analysis and dosimetric parameters

In the present study, a clinician manually delineated the spleen on each planning-CT scan (level: 40HU, window: 400HU) and MRI (level: 175, window: 350 arbitrary units). Contoured splenic volumes from both modalities were compared. For 10 randomly selected patients, a second investigator generated splenic contours, and Dice indices were calculated. Splenic dose-volume histograms (DVH) were computed using the original baseline plan dose distribution. The MSD, spleen Dmax (=D_0.03cc_), and spleen V_x_ (relative volume receiving ≥ x Gy) for 5–10-20–30 Gy levels were extracted. All reported doses are in physical doses, and GTV and PTV values were derived from baseline contours.

To study post-SABR alterations, the spleen was delineated on follow-up diagnostic CT scans at 6, 12 and 24 months (±2 months) using standard settings. SV changes were calculated as relative percentage variation compared to baseline on the simulation CT (<0 % means reduction, >0 % means increase). As SV can vary due to factors including meals, breath-hold, stress, medication and comorbidity, we selected a 20 % SV change threshold to define significant changes over time [[19], [20], [21]]. For splenic delineation and analysis, all imaging and dosimetric data were imported into Velocity v4.1 (Varian Medical Systems, Inc.).

### 2.4 Planning re-optimization for selected patients

For 4 patients with the highest MSD in our cohort, we aimed to assess whether stricter dosimetric goals (MSD < 10 Gy, splenic V10 as low as possible) could have been achieved, as a third-order priority after radiosensitive OAR protection and PTV coverage. Target coverage was evaluated by quantifying the Dmean and V95% of GTV and PTV. Institutional OAR constraints to the stomach, bowel, and duodenum were based on the evaluation of the V36Gy, V33Gy, and V25Gy, which should be lower than 0.1, 1.0, and 5 cm3, respectively [17]. Also, planning constraints for the ipsilateral kidney require that two-thirds of the renal volume should not receive a dose exceeding 18 Gy [22]. Even without specific dose constraint, we included in our analysis the dose received by the vertebral bodies adjacent to the PTV (as a single volume up until one level above and below the PTV).

### 2.5 Statistical analysis

Continuous variables were compared with Wilcoxon’s rank-sum test, and categorical variables using Fisher’s exact test. Longitudinal evolution within the same individuals was analyzed with Wilcoxon matched-pairs signed-rank tests. A significance level of p < 0.05 was applied. Univariable and multivariable linear regression (LinR) analyses explored associations between baseline characteristics and MSD, as well as between SV changes and splenic dose parameters. The threshold of 20 % reduction in SV change (yes vs. no) was used as binary outcome for logistic regression (LogR) analyses, providing odds ratios (ORs). For V5Gy and V10Gy, ORs were expressed as ratio per 10 % increase. Parameters with p < 0.10 in univariable analyses were included in multivariable regression models. Because of collinearity, spleen dosimetric parameters were added individually to separate multivariable models; for other parameters the reference was based on a model that included the MSD. All analyses were conducted using R (v4.2.2) and RStudio (v2022.12.0 + 353).

## 3 Results

### 3.1 Patient characteristics

A total of 106 patients, with 113 adrenal tumors, were eligible for study (Table 1). The median age was 66 years and 56 % of patients had left-sided adrenal tumors. Concurrent systemic therapy (defined as ≤3 months before/after SABR) was administered in 51 %. Median GTV and PTV were 25.1 cc (range 1.3–252.1) and 40.0 cc (range 3.6–319.2).

**Table 1:** Patient and tumor characteristics of 113 adrenal metastases treated in 106 eligible patients.

Characteristic	n (%) or median (range)
Age	66 (41–90)
Sex	
Male	71 (67 %)
Female	35 (33 %)
ECOG	
0–1	81 (76 %)
2–4	22 (21 %)
Not reported	3 (3 %)
Histology	
Non-small cell lung	72 (68 %)
Renal	7 (7 %)
Colon	5 (5 %)
Melanoma	4 (4 %)
Other	18 (16 %)
Timing of adrenal metastases	
Synchronous	17 (15 %)
Metachronous	44 (39 %)
Oligoprogressive	51 (45 %)
Oligopersistant	1 (1 %)
Metastatic burden	
Solitary	44 (39 %)
Oligometastatic (≤5)	62 (55 %)
Multimetastatic	7 (6 %)
Tumor laterality	
Left	63 (56 %)
Right	50 (44 %)
Number of fractions	
1	16 (14 %)
3	21 (19 %)
5	72 (64 %)
Other	4 (3 %)
GTV size (cc)	25.1 (1.3–252.1)
PTV size (cc)	40.0 (3.6–319.2)
Spleen volume (cc)	182.3 (64.8–685.6)
Systemic therapy during or within 3 months preceding/after SABR	
Yes	58 (51 %)
Immunotherapy	33 (29 %)
Chemotherapy	21 (19 %)
Other	6 (5 %)
No	55 (49 %)

The commonest SABR scheme used was 50 Gy in 5 fractions (53 %, BED_10_ 100 Gy). Single fraction treatment was sometimes used, predominantly for right-sided adrenal tumors. Otherwise, baseline characteristics were similar between left- and right-sided adrenal tumors.

Follow-up CT scans were accessible at 6, 12 and 24 months for 59 adrenals (including 33 left adrenals), 47 adrenals (including 27 left adrenals), and 31 adrenals (including 20 left adrenals), respectively. Initial SV and GTVs were similar regardless of tumor laterality or receipt of systemic treatments. Baseline clinical and treatment characteristics were generally balanced between right- and left-sided populations across timepoint (6, 12 and 24 months), except at 12 months (n = 47) where concurrent or subsequent systemic therapy use was more frequent in the right-sided group (90.0 % vs. 59.3 %, p = 0.02), mainly due to higher chemotherapy use (50.0 % vs. 22.2 %, p = 0.065). For both right- and left-sided adrenals, immunotherapy use was comparable (60 % versus 44.4 %, p = 0.38). A similar trend was observed at 24 months (n = 31) with more frequent use of systemic therapy in right-sided patients (100 % vs. 65.0 %, p = 0.03), including higher, though non-significant, rates of immunotherapy and chemotherapy.

### 3.2 Splenic dosimetric parameters in the overall cohort

Median baseline SV on CT was 182.3 cc (range 64.8–685.6) and 183.4 cc (range 66.0–786.9) on MRI. Dice indices for spleen delineation between two independent observers on CT of 10 random patients demonstrated scores > 0.95, indicating good inter-observer agreement.

Median MSD, V5Gy and V10Gy for left-sided adrenal plans were 9.7 Gy (range 1.5–28.4), 63.4 % (range 4.4–100.0 %) and 46.3 % (range 0–100 %), respectively. Corresponding values for right-sided adrenal plans were 1.5 Gy (range 0.2–5.9), 0.1 % (range 0–55.5 %) and 0 % (range 0–6.2 %). The 10 Gy MSD threshold was exceeded in 28 cases (25 %); all in left-sided adrenal tumors (i.e. 44 % of left adrenal plans). GTV was significantly larger in patients with MSD > 10 Gy than MSD ≤ 10 Gy (58.3 cc vs. 25.2 cc, *p* = 0.003), but baseline SV was not significantly different (194.7 cc vs. 219.5 cc, *p* = 0.21). In both univariable- and multivariable analyses, MSD was significantly associated with lesion laterality (*p* < 0.001), prescription dose (*p* = 0.02), and GTV (*p* < 0.001) (Supplementary Table 1). Left-sided treatments yielded significantly higher splenic dosimetric parameters (*p* < 0.001, Supplementary Table 2).

### 3.3 Post-treatment spleen volume changes

**Fig. 1:**
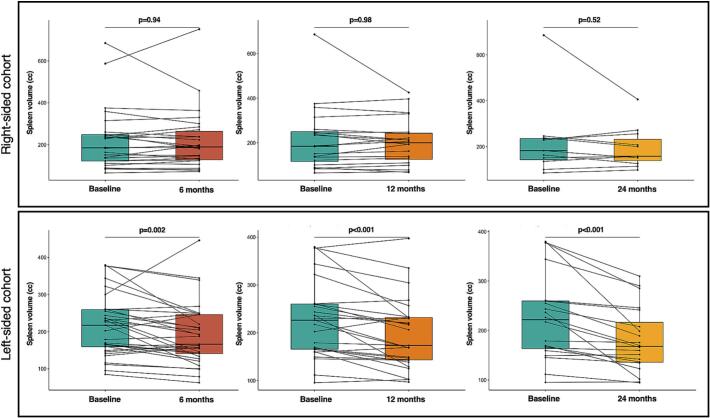
Spleen volume changes for right-sided *(upper panel)* and left-sided tumors *(lower panel)* in the group and for each patient individually at 6, 12 and 24 months.

Left-sided SABR was associated with significantly larger mean SV reductions compared to right-sided SABR at 6 months (−10.5 % vs + 0.7 %, *p =* 0.02) and 12 months (−12.0 % vs + 0.6 %, *p =* 0.006), with a similar trend at 24 months (−17.3 % vs −3.1 %, *p =* 0.06). Pairwise comparisons revealed significantly reduced SV in patients with left-sided tumors at 6/12/24-month intervals, but not for patients with right-sided tumors (Fig. 1).

**Fig. 2:**
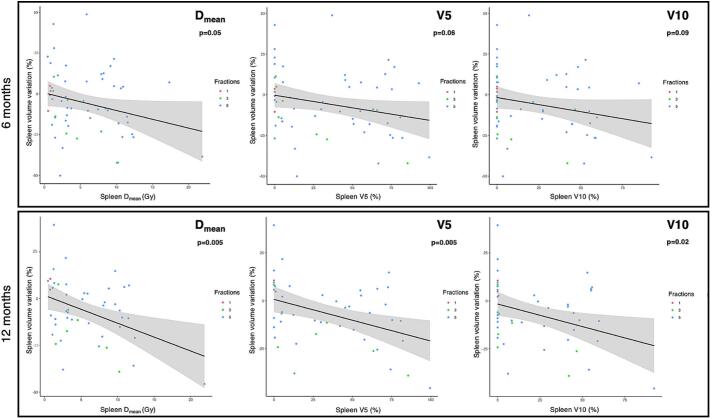
Linear regression models of splenic volume variations at 6- (top) and 12- (bottom) months according to the mean spleen dose, spleen V5 and V10. Point colors indicate the number of fractions (red = 1, green = 3, blue = 5). Shaded areas represent 95 % confidence intervals.

In the subgroup of patients with available follow-up CT scans, patients with an MSD > 10 Gy were exclusively treated for left-sided adrenals, all using a 5-fraction scheme except one patient that was treated in 3 fractions. For patients with an MSD > 10 Gy, a > 20 % decrease in SV was observed in 46 % of evaluable patients at 6 months, 40 % of patients at 12 months and 50 % at 24 months. Conversely, an MSD > 10 Gy was delivered to 50 % of evaluable patients with SV reduction > 20 % at 6 months, 40 % of patients at 12 months, and 33 % of patients at 24 months. When using a SV reduction threshold of > 20 % during follow-up, univariable logistic regression analysis revealed the MSD and V5Gy to be associated with SV shrinkage at 6 months. In multivariable analysis, the baseline SV (OR 1.01, p = 0.046), MSD (OR 1.19, p = 0.033), V5Gy (OR 1.36, p = 0.017) and V10Gy (OR 1.32, p = 0.046) were significantly related to a SV reduction of > 20 %. The MSD, V5Gy and V10Gy were also associated with the reduction of SV at 12 months in univariable analysis. After adding the baseline SV to the multivariable model, MSD (OR 1.24, p = 0.03), V5Gy (OR 1.42, p = 0.02) and V10Gy (OR 1.44, p = 0.03) remained significant. For both timepoints, V20Gy and V30Gy were not significantly associated with SV decrease (Supplementary Table 3). Linear regression models assessing SV changes at 6 and 12 months as a function of the MSD, V5 and V10 in the overall population are presented in Fig. 2.

### 3.4 Re-planning optimization

To assess whether initial plans could be optimized with regards to spleen incidental doses, we selected four patients among highest MSD in our cohort and performed an optimized Linac-MR replanning with the following objectives: MSD < 10 Gy, spleen V10 as low as possible. All four patients were planned for 50 Gy in 5 fractions to the left adrenal. Initial MSD ranged from 17.0 to 21.9 Gy, and spleen V10 from 80.2 % to 92.2 %. After replanning, optimized MSD ranged from 9.8 to 10.1 Gy and optimized spleen V10 from 33.6 % to 42.6 %. PTV coverage target as well as other organs at risks’ institutional constraints (bowel, duodenum, stomach, kidney) were still met, and vertebral doses remained in the same range. A small increase in digestive doses (especially for stomach low doses) was observed, however significantly under the constraint limit. An example of replanning for one patient (initial and optimized plans, dose-volume histogram) can be found in Supplementary Fig. 1, and details about initial and optimized plans for all four patients are described in Supplementary Table 4.

## 4 Discussion

Our single-institutional study of patients treated with adrenal MR-SABR revealed that 44 % of left-sided treatments resulted in an MSD > 10 Gy, which in turn was associated with a subsequent reduction of >20 % in SV for 40–50 % of those patients in the first year after SABR. We observed associations between SV reduction at 6–12 months and mean splenic dose, V5Gy and V10Gy. Late SV decreases were not observed after right-sided adrenal SABR with minimal splenic exposure, despite exposure to other factors such as systemic therapies. Apart from the use of more single-fraction SABR schemes for right-sided tumors, baseline patient and treatment characteristics were similar between groups.

The implications of the observed decreases in SV post-SABR are uncertain. Contradictory findings between SV and function have been reported [11,14]. While the diagnosis of hyposplenism involves evaluating the filtering function of the red pulp [23] or monitoring changes in IgM memory B cells [24], such data were unavailable for our patients. A 37 % mean reduction in splenic volume at 4-years and an increased risk of pneumonia were found after unintentional splenic irradiation during large-field adjuvant chemo-radiotherapy in gastric carcinoma [11]. Studies have reported correlations between splenic dose volume parameters and severe lymphopenia [[6], [7], [8], [9], [10], [11], [12], [13], [14]]. For instance, Ma et al. found that MSD strongly predicts radiation-induced lymphopenia with each 1-Gy increase significantly raising the risk, and Romano et al., studying patients with pathological spleens (splenomegaly), identified spleen V_5Gy_ as an independent predictor of minimum absolute lymphocyte count and survival outcomes [25,26]. In 132 patients with pancreatic tumors who were treated with 5-fraction SABR, a spleen V_10Gy_ threshold of 4.17 % was found to predict grade-2 lymphopenia [27]. Childhood cancer survivors exposed to high splenic doses are at risk of late infections [28,29]. However, our patients had undergone extensive systemic treatments including immunotherapy, and early post-SABR therapies, which prevents reliable conclusions to be drawn from lymphocyte counts [30].

Recent guidelines recommend MSD < 10 Gy for use in conventional radiotherapy, with some proposing more stringent constraints to reduce radiation-induced lymphopenia [15,16,31]. The CORSAIR initiative outlines constraints for the spleen, suggesting MSD < 8.8 Gy, V_5-10Gy_ < 30 %, and V_15-20Gy_ < 20 % [32]. However, awareness of these constraints is low among treating physicians [16]. When significant spleen irradiation is unavoidable, discussions regarding prophylactic vaccination, education, and antibiotic prophylaxis are crucial [15].

In the last part of our results, we showed that more advantageous spleen dose parameters could be achieved in a population where loose spleen dose constraints were routinely applied. However, as the adage goes, “dose has to go somewhere”, and reducing spleen dose parameters was associated with a small increase in digestive doses, however still well below institutional thresholds. Our goal was mostly to illustrate that optimization could be feasible when the spleen was brought in the overall equation, but should not impact the foremost protection of other radiosensitive OAR or PTV coverage solely based on our findings.

Limitations of our study include the fact that dosimetric analyses were based on the initial SABR plans at simulation, which only approximates the actual spleen doses given the use of a daily adaptive workflow. This choice was driven by the absence of reliable systematic dose accumulation workflow and validated model that would allow for accurate deformable dose mapping and reconstruction in our cohort. Moreover, while near-maximum doses or higher isodoses may be affected, differences in mean organ doses, especially for fixed or peripheral structures like the spleen, are often within a limited range and initial plan data as a surrogate appeared an acceptable approach. Our population was also heterogenous in terms of primary tumor histology, systemic therapy and metastatic disease presentation, all of which which may influence SV [33,34]. Finally, we included various fractionation schemes in our analysis, and reported physical doses only. While the true alpha/beta ratio of the spleen remains uncertain, MSD of around 10 Gy in 5 fractions, which was most of patients with MSD > 10 Gy, is equivalent to 10 Gy in Equivalent Dose in 2 Gy Fractions (EQD2, a/b = 3).

In conclusion, unlike for right-sided adrenal metastases, a mean spleen dose of > 10 Gy is frequently encountered in SABR for left-sided adrenal metastases. In those patients, significant SV reductions were observed in 40–50 % of patients within 1-year after SABR, and SV decreases were associated with other splenic dose parameters such as the V5 and V10. This finding warrants further investigation and emphasizes the need to better understand splenic dosimetry and its effects in upper-abdominal SABR.

## CRediT authorship contribution statement

**Nicolas Giraud:** Conceptualization, Data curation, Formal analysis, Investigation, Methodology, Writing – original draft. **Miguel A. Palacios:** Investigation, Methodology, Supervision, Writing – review & editing. **John R. van Sornsen de Koste:** Data curation, Formal analysis, Investigation, Writing – review & editing. **Antonio M. Marzo:** Data curation, Writing – review & editing. **Peter S.N. van Rossum:** Formal analysis, Investigation, Writing – review & editing. **Famke L. Schneiders:** Conceptualization, Methodology, Supervision, Writing – review & editing. **Suresh Senan:** Conceptualization, Methodology, Supervision, Writing – review & editing.

## Funding

None.

## Declaration of competing interest

The authors declare the following financial interests/personal relationships which may be considered as potential competing interests: N.G., M.P., J.S.K, A.M and P.V.R have declared that they have no known competing financial interests or personal relationships that could have appeared to influence the work reported in this paper. S.S. and F.S. have received honoraria and departmental research funding from ViewRay.

## Data Availability

Research data are stored in an institutional repository and will be shared upon request to the corresponding author.
