# Supplementary material for: Changes in splenic volumes following stereotactic ablative radiotherapy (SABR) to adrenal tumors
Source: Clin Transl Radiat Oncol. 2025 Jul 7;54:101011. doi: 10.1016/j.ctro.2025.101011 (PMC12275063; doi:10.1016/j.ctro.2025.101011)

**Supplementary Table 1.** Association between the MSD and baseline characteristics in univariable and multivariable linear regression analysis.

|  | Univariable analysis | | Multivariable analysis | |
| --- | --- | --- | --- | --- |
|  | β coefficient (95%CI) | p-value | β coefficient (95%CI) | p-value |
| Age |  | 0.87 |  |  |
| Sex (reference: Female) |  | 0.40 |  |  |
| ECOG (reference: 0) |  | NS for all categories |  |  |
| Treatment side (reference: Left) | -8.0 (-9.4 to -6.5) | **<0.001** | -7.7 (-9.1 to -6.3) | **<0.001** |
| Histology (reference: NSCLC) |  | NS for all categories |  |  |
| Metastatic timing (reference: Synchronous) |  | NS for all categories |  |  |
| Metastatic burden (reference: Solitary lesion) |  | NS for all categories |  |  |
| Prescription dose | 0.12 (0.02 to 0.22) | **0.015** | 0.07 (0.01 to 0.14) | **0.023** |
| GTV | 0.03 (0.01 to 0.05) | **0.014** | 0.03 (0.02 to 0.05) | **<0.001** |
| Baseline spleen volume |  | 0.22 |  |  |
| Concurrent or sequential IO (reference: No) |  | 0.71 |  |  |
| Concurrent or sequential ChT (reference: No) |  | 0.29 |  |  |

**Supplementary Table 2.** Splenic dose parameters according to laterality of adrenal tumor (MSD = mean spleen dose)

|  | Left adrenal gland (n=63) | Right adrenal gland (n=50) |
| --- | --- | --- |
| MSD (Gy), median (range) | 9.7 (1.5-28.4) | 1.5 (0.2-5.9) |
| MSD >10Gy, n (%) | 28 (44.4) | 0 (0.0) |
| D_0.03 cc_ (Gy), median (range) | 43.9 (9.2-66.1) | 5.2 (0.8-17.7) |
| V_5Gy_ (%), median (range) | 63.4 (4.4-100.0) | 0.1 (0.0-55.5) |
| V_10Gy_ (%), median (range) | 46.3 (0.0-100.0) | 0.0 (0.0-6.2) |
| V_20Gy_ (%), median (range) | 7.8 (0.0-81.9) | 0.0 (0.0-0.0) |
| V_30Gy_ (%), median (range) | 0.8 (0.0-34.0) | 0.0 (0.0-0.0) |

**Supplementary Table 3**. Univariable (UVA) and multivariable (MVA) logistic regression analyses of factors associated with spleen volume reduction >20% at 6 months (n=59) and 12 months (n=47) follow-up, based on baseline and dosimetric parameters.

|  | Spleen volume variation at 6 months | | Spleen volume variation at 12 months | |
| --- | --- | --- | --- | --- |
|  | UVA  p-value (OR (95%CI)) | MVA  p-value (OR (95%CI)) | UVA  p-value (OR (95%CI)) | MVA  p-value (OR (95%CI)) |
| Age | 0.16 (0.95 (0.89-1.02)) |  | 0.80 (1.01 (0.94-1.09)) |  |
| Sex (reference: Female) | 0.35 (0.54 (0.14-2.09)) |  | 0.99 (0.99 (0.23-5.23)) |  |
| Metastatic burden (reference: Solitary lesion) Oligometastatic  Multimetastatic | 0.08 (4.3 (0.97-30.4))  0.23 (5.5 (0.2-94.9)) | 0.14 (3.63 (0.73-27.12))  0.13 (9.53 (0.33-188.10)) | 0.12 (3.78 (0.81-27.47)) |  |
| Pathology (reference: NSCLC) | NS for all categories |  | NS for all categories |  |
| Prescription dose | 0.24 (0.96 (0.90-1.03)) |  | 0.82 (0.99 (0.92-1.09)) |  |
| GTV | 0.93 (1.00 (0.98-1.02)) |  | 0.83 (1.00 (0.97-1.03)) |  |
| Baseline spleen volume | 0.064 (1.01 (1.00-1.01)) | **0.046** (1.01 (1.00-1.01)) | 0.11 (1.01 (1.00-1.01)) | 0.07 (1.01 (1.00-1.01)) |
| Concurrent or sequential IO (reference: No) | 0.56 (1.46 (0.41-5.56)) |  | 0.43 (0.57 (0.13-2.31)) |  |
| Concurrent or sequential CT (reference: No) | 0.65 (0.74 (0.18-2.70)) |  | 0.66 (1.39 (0.31-5.83)) |  |
| MSD | **0.045** (1.15 (1.01-1.34)) | **0.033** (1.19 (1.03-1.42)) | **0.033** (1.20 (1.03-1.46)) | **0.029** (1.24 (1.04-1.54)) |
| V5Gy | **0.032** (1.26 (1.03-1.59)) | **0.017** (1.36 (1.08-1.80)) | **0.019** (1.36 (1.07-1.83)) | **0.018** (1.42 (1.09-2.00)) |
| V10Gy | 0.064 (1.26 (0.99-1.64)) | **0.046** (1.32 (1.02-1.77)) | **0.032** (1.38 (1.04-1.92)) | **0.029** (1.44 (1.06-2.06)) |
| V20Gy | 0.15 (1.54 (0.85-3.00)) |  | 0.11 (1.84 (0.94-4.51)) |  |
| V30Gy | 0.17 (11.6 (0.76-1141.2)) |  | 0.20 (27.3 (0.98-13094.6)) |  |

Abbreviations: OR=odds-ratio, CI=confidence interval, NSCLC=non-small-cell lung cancer, GTV=gross tumor volume, IO=immunotherapy, CT=chemotherapy, MSD=mean spleen dose.

**Supplementary Table 4**. Initial and replanned dose parameters after spleen dose optimization for 4 patients treated with 50 Gy in 5 fractions to the left adrenal.

|  | | Patient 1 | | Patient 2 | | Patient 3 | | Patient 4 | |
| --- | --- | --- | --- | --- | --- | --- | --- | --- | --- |
|  |  | Initial | Opt | Initial | Opt | Initial | Opt | Initial | Opt |
| Spleen | MSD_(Gy)_ | 21.9 | 10.1 | 17.5 | 10.0 | 17.3 | 9.8 | 17.0 | 9.8 |
|  | V5_(%)_ | 99.4 | 67.6 | 97.6 | 71.4 | 92.7 | 70.3 | 94.6 | 60.2 |
|  | V10_(%)_ | 92.2 | 36.7 | 88.6 | 33.6 | 84.7 | 41.0 | 80.2 | 42.6 |
|  | V20_(%)_ | 56.0 | 9.8 | 25.3 | 10.9 | 32.9 | 6.5 | 27.2 | 10.9 |
| GTV | Mean_(Gy)_ | 55.5 | 57.0 | 53.1 | 55.8 | 53.8 | 55.2 | 55.4 | 57.5 |
|  | D95_(Gy)_ | 50.0 | 54.6 | 41.5 | 43.7 | 49.8 | 50.9 | 50.6 | 53.9 |
| PTV | Mean_(Gy)_ | 53.7 | 55.9 | 51.3 | 53.6 | 52.9 | 54.1 | 53.7 | 56.3 |
|  | D95_(Gy)_ | 46.8 | 51.7 | 34.0 | 36.5 | 47.2 | 47.5 | 47.4 | 50.3 |
| Stomach | V25_(cc)_ | 2.6 | 4.9 | 3.8 | 8.4 | 3.2 | 18.8 | 2.6 | 1.4 |
|  | V33_(cc)_ | 0 | 0.2 | 0.01 | 0.09 | 0 | 0.8 | 0 | 0.1 |
|  | V36_(cc)_ | 0 | 0.02 | 0 | 0 | 0 | 0 | 0 | 0.02 |
| Duodenum | V25_(cc)_ | 0 | 0 | 0 | 0 | 0.3 | 1.0 | 0 | 0 |
|  | V33_(cc)_ | 0 | 0 | 0 | 0 | 0 | 0 | 0 | 0 |
|  | V36_(cc)_ | 0 | 0 | 0 | 0 | 0 | 0 | 0 | 0 |
| Bowel | V25_(cc)_ | 0.4 | 0 | 3.5 | 7.3 | 0.2 | 1.2 | 0 | 0 |
|  | V33_(cc)_ | 0 | 0 | 0.03 | 0.3 | 0 | 0 | 0 | 0 |
|  | V36_(cc)_ | 0 | 0 | 0 | 0.015 | 0 | 0 | 0 | 0 |
| Left kidney | V18_(%)_ | 9.7 | 13.5 | 25.1 | 24.5 | 18.3 | 18.1 | 15.9 | 10.9 |
| Vertebra | V5_(cc)_ | 125.4 | 80.6 | 93.0 | 65.6 | 144.9 | 137.7 | 101.9 | 77.2 |
|  | V10_(cc)_ | 100.2 | 61.0 | 49.9 | 23.9 | 103.7 | 104.6 | 93.3 | 57.4 |

**Supplementary Figure 1**. Initial and spleen-optimized plans for a left adrenal metastasis treated with MR-SABR delivered in 5 fractions of 10Gy.


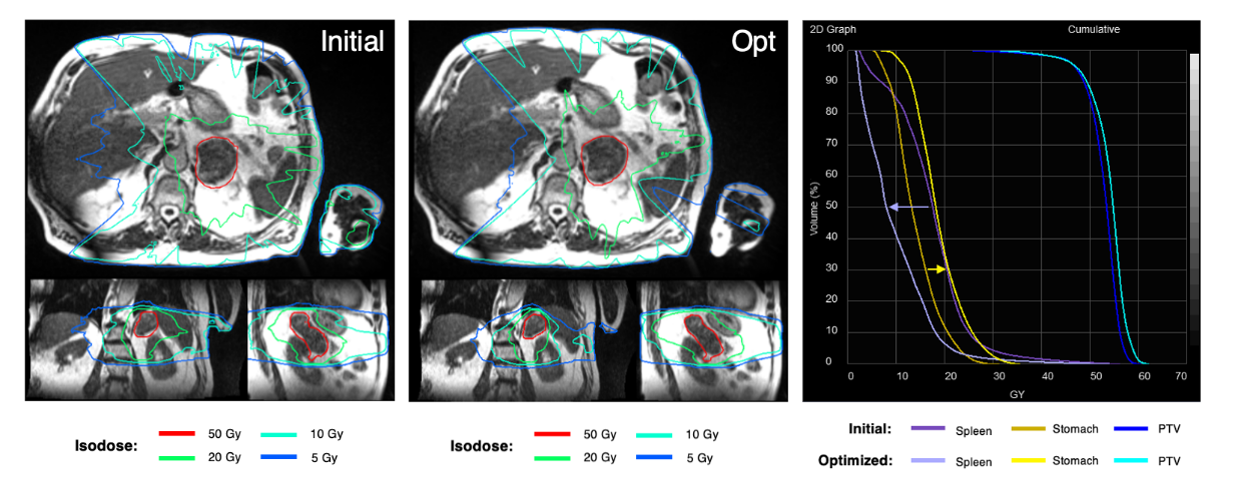

Supplement: Supplementary Data 1 [file mmc1.docx]
